# Clinical characteristics and outcome of cardiac resynchronization therapy for heart failure in National Hospital of Sri Lanka from year 2005 to 2020 – a retrospective observational study

**DOI:** 10.1186/s12872-024-03719-z

**Published:** 2024-01-23

**Authors:** Sandun Prabath Iddagoda Hewage Don, Kuruppumullage Chamini Nuwansika Kuruppu, Asunga Dunuwille, Asanka Wijewardena, Rohan Gunawardena

**Affiliations:** Cardiac Electrophysiology Unit, Institute of Cardiology, National Hospital of Sri Lanka, Colombo, Sri Lanka

**Keywords:** Cardiac Resynchronization Therapy, Heart Failure, CRT

## Abstract

**Background:**

Cardiac resynchronization therapy (CRT) has been a well-established treatment modality for moderate to severe left ventricular systolic dysfunction with left ventricular desynchrony. This is the pioneering study that analyses the cohort who underwent CRT implantation at the National Hospital of Sri Lanka (NHSL) in Colombo from 2005 to 2020.

**Objectives:**

This study was carried out to describe socio-demographic factors, improvements in clinical outcome (symptoms, electrocardiographic, and echocardiographic features), and post-CRT complications in the study population, as well as to determine the efficacy of CRT in heart failure.

**Method:**

A retrospective observational study was conducted on all the living patients who had undergone CRT implantation from 2005 to 2020. The data was gathered from all the consented patients who visited the device programming clinic using a physician-administered questionnaire and clinical records. Data was analyzed using SPSS 25, and significant statistics were assessed with the McNemer test, the Student T test, and the Chi-Squared test.

**Results:**

The study included 50 patients with a mean age of 52.82+/− 11.66 years and female predominance (56%, *n* = 28). Idiopathic dilated cardiomyopathy (50%, *n* = 25) was the leading etiological factor, followed by ischemic cardiomyopathy (28%, *n* = 14). Clinical symptoms have improved significantly with CRT implantation (*p* < 0.001). A significant improvement was found in NYHA functional class (*p* < 0.001, 95% CI = 0.072 to 0.284), QRS width (*p* < 0.001, 95% CI = 0.229 to 0.534), ejection fraction (*p* < 0.001, 95% CI = − 16.437 to − 8.504), and LV EDD (p < 0.001, 95% CI = 2.89 to 9.24). Post-CRT complications included lead malfunction (6%, *n* = 3) and chronic (14%, *n* = 7), bleeding or hematoma (2%, *n* = 1), pocket erosion or infection (6%, *n* = 3), and infective endocarditis (2%, *n* = 1).

**Conclusion:**

According to the study, CRT significantly improves both clinical and functional outcomes in patients with moderate to severe heart failure.

## Introduction

Heart failure (HF) is defined as a complex clinical syndrome, which results from any structural or functional disorder that impairs the ability of the ventricle to fill or eject blood, characterized by specific symptoms such as orthopnea and dyspnea, and signs such as edema [1]. With the prolonged survival of the aging population and the prolongation of lives of cardiac patients by modern therapeutic modalities, heart failure prevalence is on the rise [2]. The etiology of heart failure can be due to a defect in pericardium, epicardium, myocardium, endocardium, heart valves or a metabolic cause. Despite the definitive management of the etiological factor, the management of heart failure always follows the same strategic principles as the pathophysiological mechanisms of heart failure are the same.

According to the international guidelines, the management of heart failure with reduced ejection fraction includes the management of the cause of HF and comorbid diseases, monitoring and prevention of further deterioration, lifestyle modifications, pharmacological therapy, cardiac rehabilitation, palliative care, device therapy, and cardiac transplantation. The devices used in heart failure include CRTs, ICDs, left ventricular assist devices, and cardiac contractility modulation therapy [3–5]. In cardiac resynchronization therapy (CRT), cardiac pacing is used in patients with left ventricular (LV) systolic dysfunction and dyssynchronous ventricular activation. The mechanism it helps to correct a failing heart is by providing simultaneous or nearly simultaneous electrical activation of the LV and right ventricle (RV) via stimulation of the LV and RV (biventricular pacing) or LV alone. The treatment for malignant ventricular arrhythmias is achieved using an implantable cardioverter defibrillator. The CRT-P can be combined with a defibrillator (CRT-D) for more effective treatment. Bi-ventricular pacing is typically done through routine intravenous RV pacing and a specifically placed LV lead through the coronary sinus branch to achieve LV pacing. Intra-cavitary LV pacing is not recommended due to technical difficulties and a high risk of systemic thrombo-embolism.

Randomized clinical trials have demonstrated that CRT reduces mortality, reduces hospitalizations, and improves functional status in patients with LVEF ≤35% and QRS duration ≥150 ms (largely with LBBB) with NYHA functional class II, III, or ambulatory IV HF [5–9]. Depending on these criteria, the response to CRT varies, with a non-respondent rate ranging from 25 to 30%. CRT devices are expensive, particularly for a resource-poor country like Sri Lanka where most patients are dependent on free health services. Therefore, proper selection of suitable candidates is very important.

There are numerous trials which have investigated CRT implantation outcome parameters in developed countries. Despite CRT implantations being in operation in Sri Lanka for more than a decade, there is no previously published data on this area. This study has been conducted to fill that deficit, and it will eventually help in improving proper selection and delivery of CRT in the Sri Lankan contest.

## Methodology

### Patient selection and eligibility criteria

A retrospective observational study was done using the details of all the living heart failure patients presented to the device programming clinic, who had undergone CRT implantation during the period of 2005 to 2020 at the National Hospital of Sri Lanka – Colombo. Ethical review committee approval was obtained from the Postgraduate Institute of Medicine (Ethical approval number: ERC/PGIM/047). A physician-administered questionnaire was used as the study instrument which consisted of 4 subsections, namely socio-demographic factors, clinical background, pre-CRT implantation (clinical, ECG and echo) assessment, post-CRT implantation assessment (clinical, ECG and echo) and complications associated with CRT implantation. The list of patients who fulfilled the eligibility criteria was identified from the patient registry and prepared according to chronological order. The patients/relatives were contacted and requested to pay a visit to the hospital after giving a brief introduction about the study verbally. At the hospital visit, prior to administration of the questionnaire, an information sheet and consent form were distributed among the subjects. The questionnaires were administered by the investigators for the consenting participants during their device programming clinic visit and clinic records were used to gather retrospective data. The data gathering was continued and finished over a 6-month period.

### Statistical analysis

All the gathered data was analyzed using descriptive statistics. Correlational statistics were used to analyze the relationship between indication and clinical improvement parameters. Considering the sample size, the statistical significance of observed parameters was determined as follows. The paired dependent qualitative variables were analyzed using the Mc Nemer test, while paired dependent quantitative variables were analyzed using the Student T test. The chi-square test was used to analyze the significance of independent paired variables. Data was analyzed using the software SPSS version 25.

## Results

The total number of participants who had undergone Cardiac Resynchronization Therapy (CRT) for heart failure during the study period (2005–2020) at NHSL Colombo and presented to follow-up clinics was 50 with a mean age of 52.82 years (standard deviation +/− 11.66). There was a female predominance (56%, *n* = 28) and the majority of patients (60%, *n* = 30) had received CRT implantation within 5 years of heart failure being diagnosed (Table 1). Diabetic Mellitus (50%, *n* = 25), hypertension (42%, *n* = 21), dyslipidemia (28%, *n* = 14) and coronary artery disease (28%, *n* = 14) were the leading comorbidities in descending prevalence with CRT implanted cohort.

**Table 1:** Baseline characteristics of the overall study population (*n* = 50)

	Frequency	Percentage (%)
Men/Women (n)	22/28	44/56
Duration of symptoms		
**•** < 5 years **•** 5–10 years **•** > 10 years	30 7 13	60 14 26
Associated comorbidities		
**•** Diabetes Mellitus **•** Hypertension **•** Dyslipidemia **•** Coronary artery disease **•** BA/COPD **•** Others (Hypothyroidism, Obesity, AF)	25 21 14 14 10 8	50 42 28 28 20 16

*BA* Bronchial Asthma, *COPD* Chronic Obstructive Pulmonary Disease, *AF* Atrial Fibrillation

The most frequent etiological factor for heart failure was idiopathic dilated cardiomyopathy (52%, *n* = 26) followed by ischemic cardiomyopathy (28%, *n* = 14) with an equal contribution from hypertension and peripartum cardiomyopathy (6%, *n* = 3) (Table 2).

**Table 2:** Etiologies for CRT Implantation RV- Right ventricular, LV- Left Ventricular

Etiology	Frequency	Percentage
Idiopathic Dilated Cardiomyopathy	26	52%
Ischemic Cardiomyopathy	14	28%
Hypertension	3	6%
Peripartum Cardiomyopathy	3	6%
High RV pacing	1	2%
Pacemaker induced heart failure	1	2%
Intermittent heart block leading to LV dysfunction	1	2%
Valvular Heart Disease	1	2%
Total	50	100%

Cardiac resynchronization therapy showed a significant clinical improvement (*p* < 0.001) in heart failure associated symptoms (Table 3).

**Table 3:** Symptomatic improvement following CRT implantation

Symptoms	Pre CRT-Percentage	Post CRT-Percentage	Mcnemer test value	95% CI
Dyspnea on exertion	96%	48%	< 0.01	0.254–4.276
Dyspnea at Rest	32%	2%	< 0.01	0.972–1.092
Orthopnea	54%	14%	< 0.01	0.593–0.926
Paroxysmal nocturnal dyspnea	42%	2%	< 0.01	0.866–1.048
Fatigue	58%	8%	< 0.01	0.745–0.997
Edema	36%	6%	< 0.01	0.678–1.025

### NYHA class

**Fig. 1:**
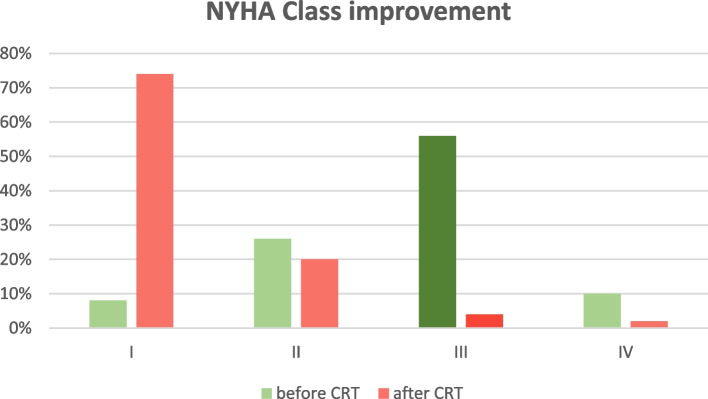
Distribution of NYHA functional class before and after CRT implantation

The majority (66%) belong to NYHA class III and IV before CRT implantation. In the assessment of NYHA class improvement, at least 1 class increment was considered as an ‘improvement’ (*n* = 42, 84%), whereas a reduction or unchanged NYHA class was considered as ‘not improved’ (*n* = 8, 16%). A significant improvement of NYHA functional class was identified with therapeutic CRT Implantation **(**Fig. 1**)** (*p* < 0.001, 95% CI = 0.072 to 0.284). EF improvement following CRT implantation in less symptomatic heart failure (NYHA class I, II) was 60% (*n* = 9) and more symptomatic heart failure (NYHA class III, IV) was 79.3% (*n* = 23). The most significant change in the NYHA Class following CRT implantation was highlighted in NYHA class III. The 56% of the study population who was belonged to NYHA class III dropped to 4% following CRT implantation.

### QRS width improvement

**Fig. 2:**
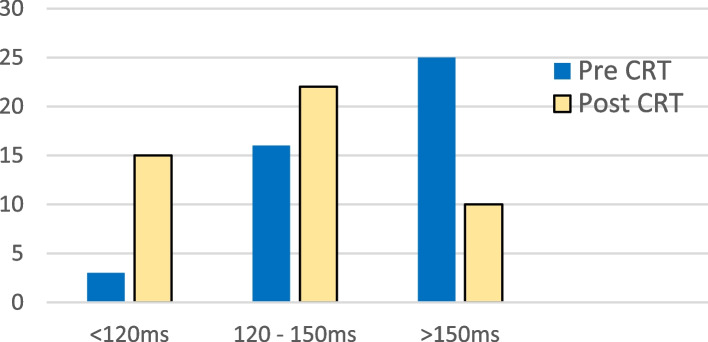
Change of QRS width by CRT implantation

The majority of patients’ QRS width was > 150 ms before CRT implantation **(**Fig. 2**)**. Out of the total study population, narrowing of QRS width by cardiac resynchronization was 59.08% (*n* = 26) while further widening of QRS or unchanged was 40.92% (*n* = 18). Overall, a statistically significant improvement in QRS width was highlighted (*p* < 0.001, 95% CI = 0.229 to 0.534).

Out of QRS width improved population (*n* = 26), EF improved in 73.07% (*n* = 19), EF did not improved in 26.92% (*n* = 7).

Out of QRS width improved, (*n* = 26), 88.4% have improved their NYHA class while 11.53% have not improved the NYHA class.

### EF improvement

The mean pre-CRT left ventricular ejection fraction of the study population was 30.91% with a standard deviation of +/− 10.28%. Following CRT implantation, the mean LV EF was improved to 43.38% with a standard deviation of +/− 13.27%. The paired sample T test highlights a significant improvement in LV ejection fraction (*p* < 0.001, 95% CI = − 16.437 to − 8.504), with a mean of 12.47% LVEF improvement with standard deviation of +/− 13.047%. There is a statistically significant (*p* = 0.01545) difference in LVEF improvement in the female subgroup (*n* = 22, 78.57%) compared to the male subgroup (*n* = 10, 45.45%), which shows a higher beneficial effect of CRT in females compared to males. In addition, LV EDD following CRT has improved significantly (*p* < 0.001, 95% CI = 2.89 to 9.24)) to mean Post CRT LVEDD 53.8+/− 12.49 mm from mean Pre CRT LVEDD 59.8+/− 10.59 mm. The average LV EDD improvement was 10.7 mm and the median LVEDD improvement was 10 mm.

### Level of recommendation of CRT implantation according to guidelines

Out of the total study population, 73.33% (*n* = 33) CRT implantations had been carried out according to class 1A guideline recommendation and 26.66% (*n* = 12) CRT implantations were not according to class 1A guideline recommendation.

Within the guideline recommended class 1A subgroup, 78.78% had LV EF improvement whereas LV EF improvement in the subgroup not belonging to class 1A was only 21.21%. This is not statistically significant as the chi-square test value was 0.170 (95% CI = 0.175 to 0.259).

### Complications

The most frequent complication seen in this study was acute (6%, *n* = 3) and chronic (14%, *n* = 7) lead malfunctions. The rest of the complications seen were bleeding/ hematoma (2%, *n* = 1), pocket erosion/ infection (6%, *n* = 3), infective endocarditis (2%, *n* = 1) in descending frequencies. No other significant complications like pneumothorax, vascular /cardiac damage were noted peri or post-operatively.

## Discussion

The Institute of Cardiology, National Hospital of Sri Lanka, Colombo has been the leading tertiary care center, which entertains referrals from all around the country as well as direct admissions. The cardiac electrophysiology service started in the year 2005. This institute has been the main service supplier for more than a decade since then. This study has bridged the gap of deficiency in CRT implantations and follow-up data by evaluating the demographics, clinical parameters and analyzing the efficacy of CRT as a heart failure treatment modality.

The mean age of developing HF in Sri Lanka was 60.66 years, and the median age was 50–59 years [10]. In contrast, the USA pooled population-based cohort median age of heart failure was 89 years (86–92) in the elderly population, and this was 76 years in Denmark (66–84) from 2010 to 2012. In the UK, the mean age of developing HF was 77.08 years [11–13]. According to the international literature, the mean age of CRT implantation in the USA was 65.4 (+/− 10.8) years, UK 63.9 ± 10.7 years and a meta-analysis done in Korea has shown a range from 58 to 72 years [1, 2, 14]. These age ranges are higher compared to the mean age of the study population, which was 52.82+/− 11.66 years. This is compatible with the published findings of early onset HF as well as life expectancy differences in Sri Lanka compared to other developed countries with higher age of HF onset and higher life expectancy.

According to census data in National Statistics in Sri Lanka, the gender distribution of the country is male 48.4% and female 51.6% [15]. This might have been a factor for this study population to have a female predominance (56%). In contrast, most of the studies done in developed countries have shown a male predominance, such as in the USA 72%, in the UK 68%, and in France 81.2% [1, 2, 16]. The beneficial effects in LVEF after CRT implantation in the study population of females have shown better values than males (females = 78.57%, males = 45.45%). This trend has been seen in a study which was conducted in the USA in 2015 [17].

The prevalence of associated comorbidities of heart failure in the CRT implanted population in Sri Lanka has shown that hypertension and diabetes mellitus were the predominant conditions. A study conducted in Canada in the year 2010 has shown a similar distribution of comorbidities in heart failure patients who had undergone resynchronization therapy, which highlights the prevalence of hypertension (52%) and diabetes mellitus (16%) [18].

A meta-analysis on cardiac resynchronization in patients with symptomatic heart failure and a study which was conducted by Mark A. Ileret et al. have shown that ischemic cardiomyopathy as the leading etiological factor for heart failure (58 and 65% respectively) [19, 20]. But in this study, most of the patients had idiopathic dilated cardiomyopathy as the major contributing factor to heart failure. This might not have affected the outcome of data, as it was shown by meta-analysis done in the USA in 2013, that CRT has similar benefits in both ischemic and non-ischemic groups [21].

There was a significant improvement in the NYHA III functional class with CRT implantation in this study population and almost a similar functional class improvements have been observed in two separate studies done in the USA in 2007, 2008 (59% improvement) and the UK in 2022 (52% improvement) [2, 22]. In contrast to subgroup analysis of NYHA functional classes in this study, Alan J. Banka et al. conducted a study in Minnesota during 2012 which shows that improvement of clinical status, LV function and size of NYHA functional class I/II CRT patients were good or better than those in NYHA functional class III/ IV [23]. A similar trend has been observed in a meta-analysis which was done by Nawaf S. Al-Majed et al. in 2011 [24].

QRS width has been considered as a surrogate marker to measure the efficacy of CRT implantation improvement. The functional class improvement of NYHA has been 88.4% for the subgroup of QRS width improved. Studies done in Canada and Korea have shown a similar beneficial effect of QRS improvement in relation to CRT efficacy [14, 25]. Sander G. Molhoek et al. in the Netherlands has observed that baseline QRS width is not a good predictor to determine the efficacy of CRT [26].

Several international studies have shown a pre-CRT LVEF of 20–30%, whereas our study has a mean pre-CRT LVEF of 30.91+/− 10.28%. This depicts a less stringent adherence of guideline recommendations for CRT implantation [6, 16, 27]. Most of the meta-analysis done in the western part of the world during the early twenty-first century proves a significant improvement in LVEF following CRT [6, 8, 28]. This study has shown similar results with significant LVEF improvement (*p* < 0.001, 95% CI = − 16.437 to − 8.504).

A significant improvement of LV EDD has been noted in a study done by William T. Abraham et al. in UK which has shown a median LV EDD improvement with CRT implantation of − 3.5 mm in patients with mean of pre-CRT LV EDD as 70 ± 10 mm comparable to the similar findings in our study which has been noted as 10 mm median LV EDD improvement in patients with 59.8+/− 10.59 mm mean pre-CRT LVEDD [8].

The rate of complications is low in this study compared to other international systematic reviews and meta-analysis done during 2003 to 2007 [6, 20, 25], but this may be due to the low population numbers.

## Limitations

The cardiac electrophysiology was a new entity in the cardiology field of Sri Lanka which was introduced in year 2005 with limited resources. It has gradually expanded over the past years, gaining new experiences. With these circumstances, there was a limitation of data which has led to a reduced total number of patients who were eligible for the study as well as reduced availability of medical records as some of them were discarded and missing. In addition, the number of implants was low, particularly in the early years as CRT was a relatively new procedure and as most patients were dependent on free devices through the National Health system, the availability of devices was also restricted. Mortality could not be assessed as planned due to the difficulty of following up patients over the past 15 years.

## Conclusion

The CRT implantations in Sri Lanka have provided significant improvements clinically, as well as in ECG and echo parameters in the study population, comparable to international results. The availability of CRT as a treatment modality for heart failure patients should be improved as it has proven benefits. Strict adherence to international criteria would further improve results, particularly in resource-poor countries.

## Data Availability

The datasets used during this study are available from the corresponding author on reasonable request.
